# Supplementary material for: A spin dynamics approach to solitonics
Source: Sci Rep. 2016 May 9;6:25685. doi: 10.1038/srep25685 (PMC4860584; doi:10.1038/srep25685)
Supplement: Supplementary Information [file srep25685-s5.pdf]

A spin dynamics approach to solitonics

Konstantinos Koumpouras,[1,*] Anders Bergman,[1] Olle Eriksson,[1] and Dmitry Yudin[2]

[1]*Department of Physics and Astronomy, Uppsala University, P.O. Box 516, 751 20 Uppsala, Sweden*
[2]*ITMO University, Saint Petersburg 197101, Russia*

SUPPLEMENTARY MOVIE LEGENDS

Supplementary movie 1. Time evolution of soliton generation. After the stabilisation of the ferromagnetic state, the the applied external magnetic field is reduced and the ferromagnetic state is still present. Afterwards an opposite external magnetic field is applied at the center of the system, strong enough to flip the spins of this region. After the flipping of the spins the opposite external magnetic field is removed. The result of the later procedure is the generation of a soliton.

Supplementary movie 2. Collision of two solitons by moving them in opposite directions, which corresponds to Fig. 4a-c. Two solitons are stabilised near the edges of our sample and two opposite spin polarised currents are applied with the same current density and non-adiabatic parameter ($j = 0.19 \times 10^{12}$ A/m^2 and $\beta = 0.2$). When solitons are close to each other we stop the flow of the spin current.

Supplementary movie 3. Annihilation of a soliton by colliding two solitons, which corresponds to Fig. 4a,d,e. The only difference of this movie compare to the previous one (movie 2) is the value of the current density which is increased in this case ($j = 0.38 \times 10^{12}$ A/m^2 and $\beta = 0.2$). Similar to the previous movie when the solitons are coming close we stop the flow of the spin current.

Supplementary movie 4. Generation of one more soliton by applying spin polarised current, which corresponds to Fig. 6. The initial position of the stabilised soliton is at the centre and in this case reversed currents are applied, the first one from the center of the soliton to the left and the second one from the center to the right. The current density for both of them is $j = 6.1 \times 10^{12}$ A/m^2 in opposite directions and non-adiabatic parameter $\beta = 0.2$. After very short time we stop the flow of the current and the perturbation which is caused by the current is big enough for the system to stabilise two solitons.

* Corresponding author. konstantinos.koumpouras@physics.uu.se
